# A Series of Spiropyrimidinetriones that Enhances DNA Cleavage Mediated by *Mycobacterium tuberculosis* Gyrase

**DOI:** 10.1021/acsinfecdis.3c00012

**Published:** 2023-02-21

**Authors:** Jo Ann W. Byl, Rudolf Mueller, Ben Bax, Gregory S. Basarab, Kelly Chibale, Neil Osheroff

**Affiliations:** † Department of Biochemistry, Vanderbilt University School of Medicine, Nashville, Tennessee 37232, United States; ‡ Medicine (Hematology/Oncology), Vanderbilt University School of Medicine, Nashville, Tennessee 37232, United States; § Drug Discovery and Development Centre (H3D), Department of Chemistry, University of Cape Town, Rondebosch 7701, South Africa; ∥ Medicines Discovery Institute, Cardiff University, Cardiff CF10 3AT, United Kingdom; ⊥ South African Medical Research Council Drug Discovery and Development Research Unit, Department of Chemistry and Institute of Infectious Disease and Molecular Medicine, University of Cape Town, Rondebosch 7701, South Africa; # VA Tennessee Valley Healthcare System, Nashville, Tennessee 37212, United States

**Keywords:** spiropyrimidinetrione, gyrase, *Mycobacterium tuberculosis*, DNA cleavage

## Abstract

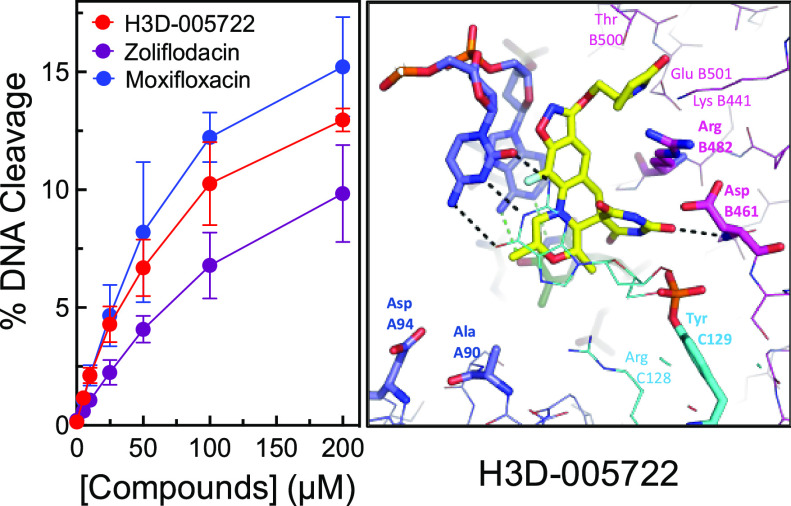

The rise in drug-resistant tuberculosis has necessitated the search for alternative antibacterial treatments. Spiropyrimidinetriones (SPTs) represent an important new class of compounds that work through gyrase, the cytotoxic target of fluoroquinolone antibacterials. The present study analyzed the effects of a novel series of SPTs on the DNA cleavage activity of *Mycobacterium tuberculosis* gyrase. H3D-005722 and related SPTs displayed high activity against gyrase and increased levels of enzyme-mediated double-stranded DNA breaks. The activities of these compounds were similar to those of the fluoroquinolones, moxifloxacin, and ciprofloxacin and greater than that of zoliflodacin, the most clinically advanced SPT. All the SPTs overcame the most common mutations in gyrase associated with fluoroquinolone resistance and, in most cases, were more active against the mutant enzymes than wild-type gyrase. Finally, the compounds displayed low activity against human topoisomerase IIα. These findings support the potential of novel SPT analogues as antitubercular drugs.

Gyrase and topoisomerase IV are the targets for fluoroquinolones, which are among the most heavily prescribed broad-spectrum antibacterials worldwide.^1−6^ These enzymes modulate the superhelical density of DNA and resolve knots and tangles in the bacterial chromosome.^4,7,8^ Fluoroquinolones inhibit the overall catalytic activities of gyrase and topoisomerase IV and stabilize covalent enzyme-cleaved DNA complexes (i.e., cleavage complexes) that are transient intermediates in the catalytic cycles of these enzymes.^1−6^ The actions of fluoroquinolones rob bacterial cells of the critical catalytic functions of gyrase and topoisomerase IV and induce enzyme-generated DNA strand breaks that trigger the SOS response and lead to cell death.^2,4−6,9^

The World Health Organization lists fluoroquinolones among the five “highest priority” and “critically important” antimicrobial classes.^10^ An important use of fluoroquinolones is the treatment of tuberculosis.^10,11^ This lung infection, which is caused by the bacterium *Mycobacterium tuberculosis*, is one of the leading causes of global mortality.^10^ The 1.5 million fatalities attributed to tuberculosis in 2020 ranked second only to COVID-19 for deaths caused by a single infectious agent.^12^

Although fluoroquinolones are used as second-line treatment for tuberculosis, members of this drug class (primarily moxifloxacin and levofloxacin) are becoming increasingly more important as a treatment for patients who have multidrug-resistant tuberculosis or are intolerant of first-line therapies.^10,11^ Unfortunately, the use of these drugs in the treatment of tuberculosis is being threatened by the rise of fluoroquinolone resistance mutations in *M. tuberculosis* gyrase, which is the only type II topoisomerase encoded by this organism.^11,13,14^

Fluoroquinolones interact with bacterial type II enzymes primarily through a water-metal ion bridge formed by a divalent metal ion that is chelated by the C3/C4 keto acid of the drug and stabilized by four water molecules.^1,15−17^ Two of these water molecules are coordinated by a highly conserved serine (originally identified as Ser83 in the GyrA subunit of *Escherichia coli* gyrase) and an acidic residue (located four positions downstream). Mutations in the residues that anchor the bridge are the most prevalent cause of fluoroquinolone resistance.^1,5,6,18,19^

In contrast to gyrase from most bacteria, *M. tuberculosis* gyrase contains an alanine (A90) in the place of the conserved serine.^20^ However, mutations in this residue and the acidic residue (D94) disrupt interactions with the water–metal ion bridge, which diminishes drug binding and causes fluoroquinolone resistance.^16^

To address the issue of fluoroquinolone resistance, two new classes of gyrase/topoisomerase IV-targeted antibacterials have been developed, novel bacterial topoisomerase inhibitors (NBTIs) and spiropyrimidinetriones (SPTs).^5,18,21^ The most clinically advanced NBTI, gepotidacin, is in phase III clinical trials for the treatment of uncomplicated urinary tract infections and uncomplicated urogenital gonorrhea.^22−25^ The most clinically advanced SPT, zoliflodacin, is in phase III clinical trials for the treatment of uncomplicated gonorrhea.^26−28^

Although far more is known about drug–enzyme interactions and drug mechanism for NBTIs than for SPTs, both classes of antibacterials appear to interact with gyrase and topoisomerase IV through residues that are not used to bind fluoroquinolones.^15,29−31^ Consequently, NBTIs and SPTs that overcome fluoroquinolone resistance have been reported.^4,18,32−35^ Furthermore, novel subsets of NBTIs and SPTs have been shown to interact with wild-type and resistant *M. tuberculosis* gyrase and overcome fluoroquinolone resistance in cultures and mouse infection models.^4,32,34,35^ Members of the novel SPT series inhibit the DNA supercoiling reaction catalyzed by wild-type *M. tuberculosis* gyrase.^34,35^ However, virtually nothing is known regarding the effects of these SPTs on the critical DNA cleavage reaction of gyrase from this organism.

Therefore, the effects of five novel SPTs^34^ on DNA cleavage mediated by wild-type and fluoroquinolone-resistant *M. tuberculosis* gyrase were assessed and compared to those of moxifloxacin, ciprofloxacin, and zoliflodacin. These SPTs displayed activities that were comparable to those of the fluoroquinolones and higher than that of zoliflodacin against the wild-type enzyme. Moreover, the SPTs maintained high activity against three of the most common fluoroquinolone-resistant mutant gyrase enzymes. These results, together with the previous cellular and in vivo data,^34,35^ suggest that SPTs may be suitable alternatives to fluoroquinolones for the treatment of tuberculosis, especially strains that carry fluoroquinolone resistance mutations in gyrase.

## Results

### Enhancement of *M. tuberculosis* Gyrase-Mediated DNA Cleavage by Novel SPTs

SPTs are an emerging class of antibacterials that target gyrase and topoisomerase IV. Current SPT drug development efforts have focused on *Neisseria gonorrhoeae* infections.^23,26−28^

**Figure 1:**
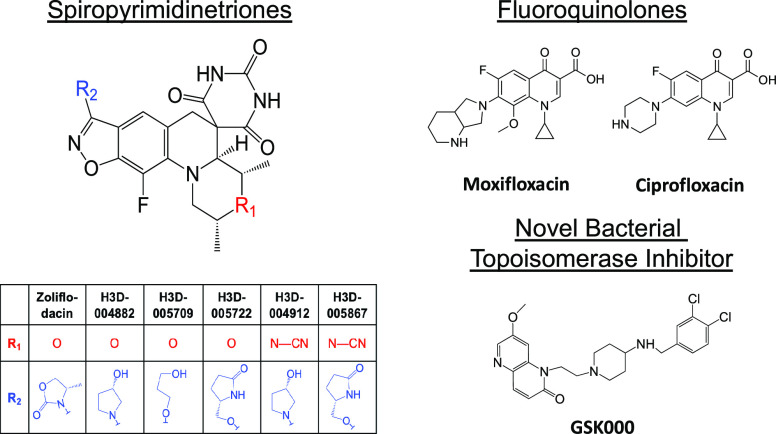
Structures of five novel SPTs, H3D-005709, H3D-004882, H3D-005722, H3D-004912, and H3D-005867, are shown. The substituents on the SPTs that vary are depicted in red (R_1_) or blue (R_2_). The structures of the SPT, zoliflodacin, the fluoroquinolones, moxifloxacin and ciprofloxacin, and the NBTI, GSK000, that were used in this study are also shown.

**Figure 2:**
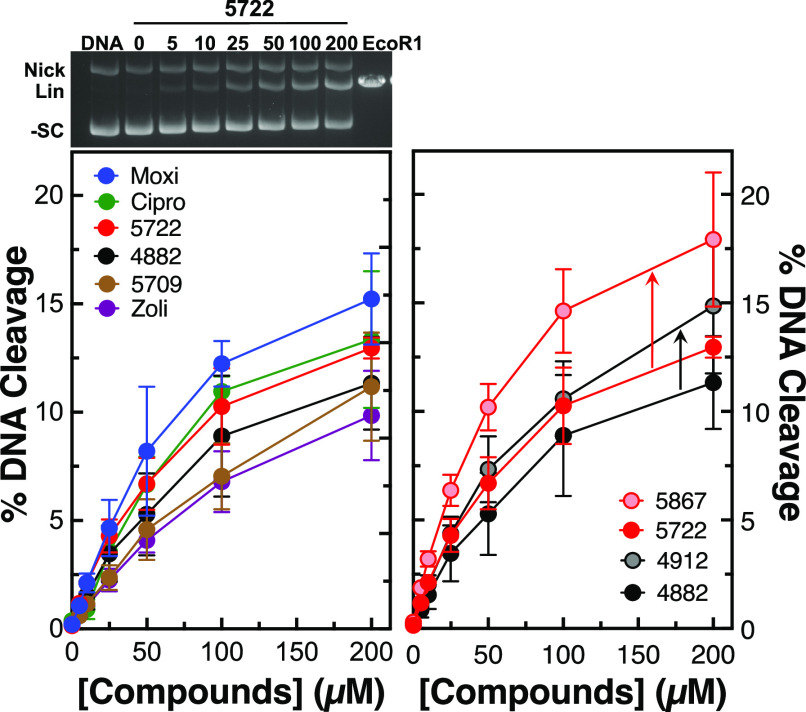
SPTs enhance double-stranded DNA breaks generated by *M. tuberculosis* gyrase. Left panel: double-stranded DNA cleavage induced by the SPTs, zoliflodacin (Zoli, purple), H3D-005709 (5709, brown), H3D-004882 (4882, black), H3D-005722 (5722, red), moxifloxacin (Moxi, blue), and ciprofloxacin (Cipro, green) is shown. Right panel: double-stranded DNA cleavage induced by H3D-004882 (4882, black) and H3D-005722 (5722, red) is compared to that of their cyano derivatives H3D-005867 (5867, salmon) and H3D-004912 (4912, silver), respectively. Error bars represent the standard deviation (SD) of at least three independent experiments. The representative agarose gel (top left) shows DNA products generated in cleavage reactions containing 0–200 μM H3D-005722. The positions of negatively supercoiled (−SC), nicked (Nick), and linear (Lin) DNA are indicated.

A recent study determined that novel SPTs also display activity against *M. tuberculosis* in cultures and mouse infection models. One of the most potent of these compounds was H3D-005722 (Figure 1, listed as compound **23** in the work of Govender et al.).^34^ H3D-005722 differs from zoliflodacin by the replacement of the 5-methyloxazolidinone group at the R_2_ position with a valerolactam with a 2-atom bridge to the benzisoxazole scaffold (Figure 1). Although members of the SPT series inhibited gyrase-catalyzed DNA supercoiling, they were not critically evaluated for their ability to enhance DNA cleavage mediated by the *M. tuberculosis* enzyme. Therefore, the effects of H3D-005722 and related SPTs (Figure 1) on the critical DNA cleavage reaction of *M. tuberculosis* gyrase were assessed (Figure 2). Results were compared to those of the fluoroquinolones moxifloxacin (which is used in the treatment of tuberculosis) and ciprofloxacin (which is heavily prescribed as a broad-spectrum antibacterial) and zoliflodacin.

The ability of H3D-005722 to induce gyrase-mediated double-stranded DNA cleavage was similar to that of moxifloxacin and ciprofloxacin and somewhat higher than that of zoliflodacin (Figure 2, left panel). The related SPTs, H3D-004882 and H3D-005709 (which differ at the R_2_ position), also induced gyrase-mediated DNA cleavage. Although their activities were slightly lower than that of H3D-005722, they were comparable to or greater than that of zoliflodacin. The activities of two additional compounds, H3D-004912 and H3D-005867, were also examined. These SPTs are analogues of H3D-00488 and H3D-005722, respectively, and replace the oxygen at R_1_ with an amino-cyano group. In both cases, the presence of the amino-cyano group enhanced the activity of the parent compound (Figure 2, right panel; see arrows).

As seen in the gel in Figure 2 (top left), H3D-005722 (and other SPTs—not shown) induced almost exclusively double-stranded DNA breaks monitored by the conversion of negatively supercoiled plasmid to linear DNA. Virtually no increase in single-stranded DNA breaks (monitored by the generation of nicked DNA molecules) was observed.

**Figure 3:**
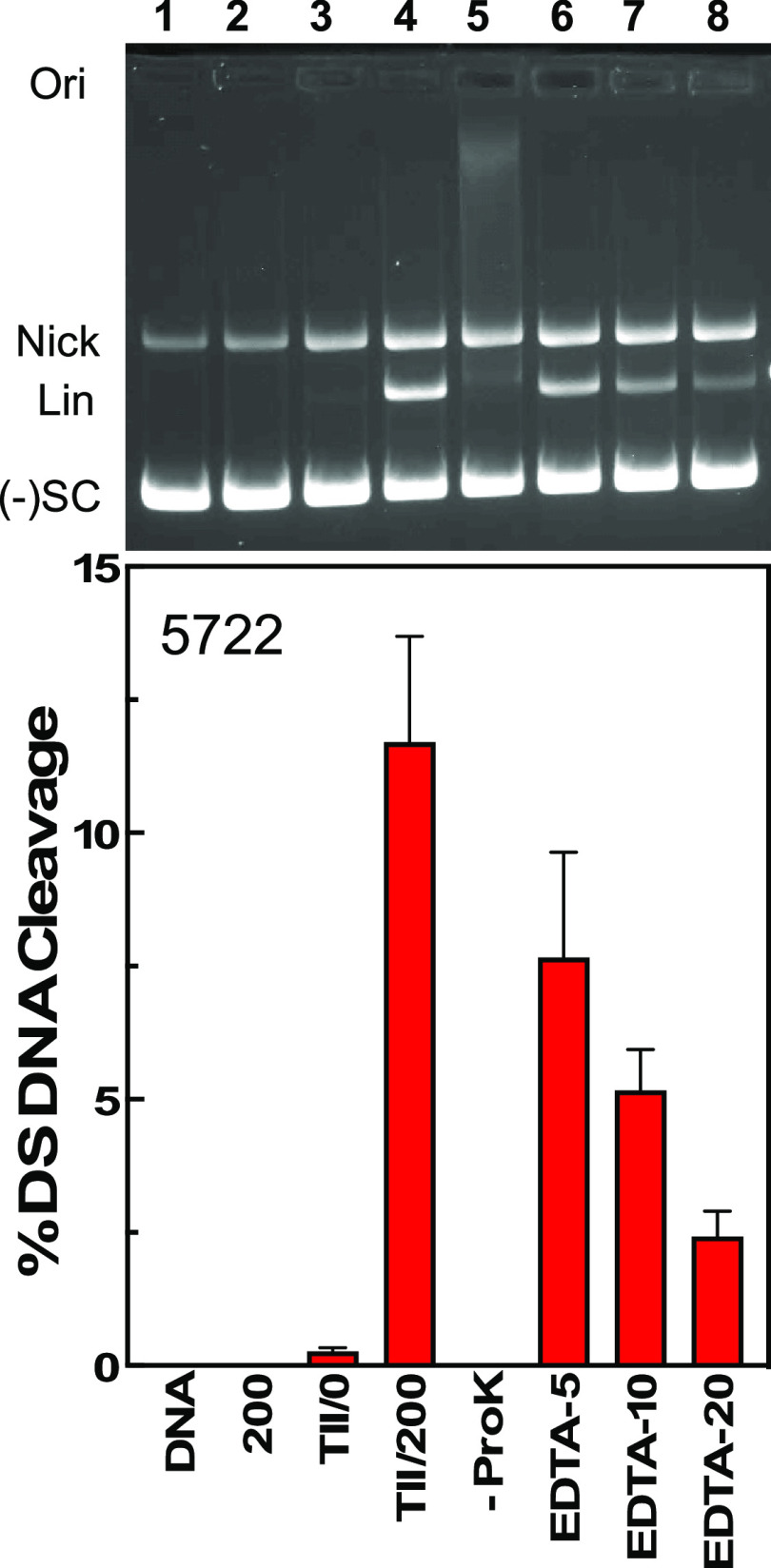
The double-stranded DNA breaks induced by H3D-005722 are mediated by *M. tuberculosis* gyrase. The graph shows reaction mixtures that contained a negatively supercoiled DNA control (DNA), negatively supercoiled DNA plus 200 μM H3D-005722 in the absence of enzyme (200), negatively supercoiled DNA plus *M. tuberculosis* gyrase in the absence of the SPT (TII/0), and a complete reaction mixture containing negatively supercoiled DNA plus 200 μM H3D-005722 and *M. tuberculosis* gyrase (TII/200). The complete reaction mixture labeled -ProK was not treated with Proteinase K to digest gyrase prior to electrophoresis. Complete reaction mixtures that were treated with EDTA for 5, 10, or 20 min prior to the addition of SDS are labeled EDTA-5, EDTA-10, or EDTA-20, respectively. Error bars represent the SD of at least three independent experiments. A corresponding representative agarose gel is shown at the top. The positions of negatively supercoiled [(−)SC], nicked (Nick), and linear (Lin) DNA as well as the gel origin (Ori) are indicated.

A series of control experiments was carried out to ensure that the enhancement of DNA cleavage seen in the presence of SPTs was mediated by gyrase, as opposed to a chemical reaction by the compounds (Figure 3). H3D-005722 was the SPT that was chosen for these experiments. Three separate results indicate that the double-stranded DNA breaks generated in the presence of H3D-005722 were generated by *M. tuberculosis* gyrase. First, no DNA cleavage was seen when 200 μM H3D-005722 was incubated with the plasmid substrate in the absence of gyrase (Figure 3, lane 2, 200). Second, because DNA cleavage products generated by gyrase are covalently attached to the protein,^16,18^ reaction mixtures must be treated with Proteinase K to digest the enzyme for cleaved DNA to run as a unique linear band. If it is not digested, protein-linked cleaved products are observed as a high molecular weight smear on the gel. As seen in lane 5 (ProK), in the absence of protease, the linear DNA cleavage product of the gyrase-containing reaction was replaced by a high-molecular-weight smear. Third, gyrase requires two active-site divalent metal ions to cleave DNA, which, in this case, is Mg^2+^.^16,18^ EDTA is able to chelate the Mg^2+^ and remove it from the enzyme only when the DNA is ligated. Consequently, treatment of gyrase-mediated reactions with EDTA prior to termination decreases the level of cleaved products over time. As seen in lanes 6–8 (EDTA-5, 10, and 20), treatment of reaction mixtures with the chelator diminished the presence of linear DNA cleavage products substantially over a time course of 5–20 min. This reversibility is inconsistent with a chemically induced DNA cleavage reaction. Taken together, these results provide strong evidence that the DNA scission observed in the presence H3D-005722 is mediated by *M. tuberculosis* gyrase.

**Figure 4:**
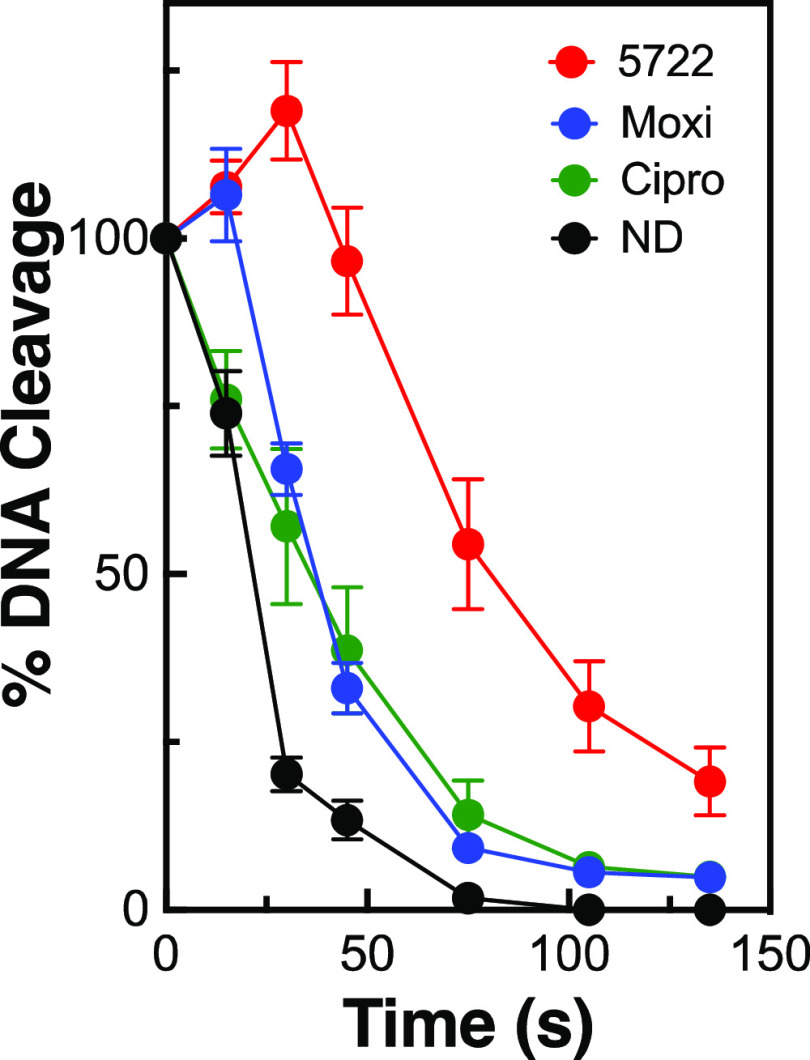
H3D-005722 inhibits DNA ligation mediated by *M. tuberculosis* gyrase. Rates of gyrase-mediated DNA ligation were monitored by the loss of double-stranded breaks in the absence of a drug (ND, black) or in the presence of 200 μM H3D-005722 (5722, red). DNA ligation assays carried out in the presence of 200 μM moxifloxacin (Moxi, blue) or ciprofloxacin (Cipro, green) are shown for comparison. Error bars represent the SD of at least three different experiments.

Compounds that raise levels of gyrase–DNA cleavage complexes can act by increasing the forward rate of DNA scission or diminishing the rate of ligation. To determine the effects of SPTs on the lifetime of cleavage complexes, a DNA ligation assay was carried out in the presence of H3D-005722. After reaction mixtures were allowed to come to cleavage–ligation equilibrium at 37 °C, ligation was induced by a shift to 75 °C (a temperature that allows DNA ligation but not cleavage).^18,36^ As seen in Figure 4, the half-life of the cleavage complex formed in the presence of H3D-005722 (∼80 s) was ∼4 times longer than that observed in the absence of the compound (∼21 s). Therefore, cleavage complexes formed in the presence of the SPT appear to be more stable than those formed in the presence of moxifloxacin or ciprofloxacin (Figure 4).

### Effects of ATP on SPT Activity

**Figure 5:**
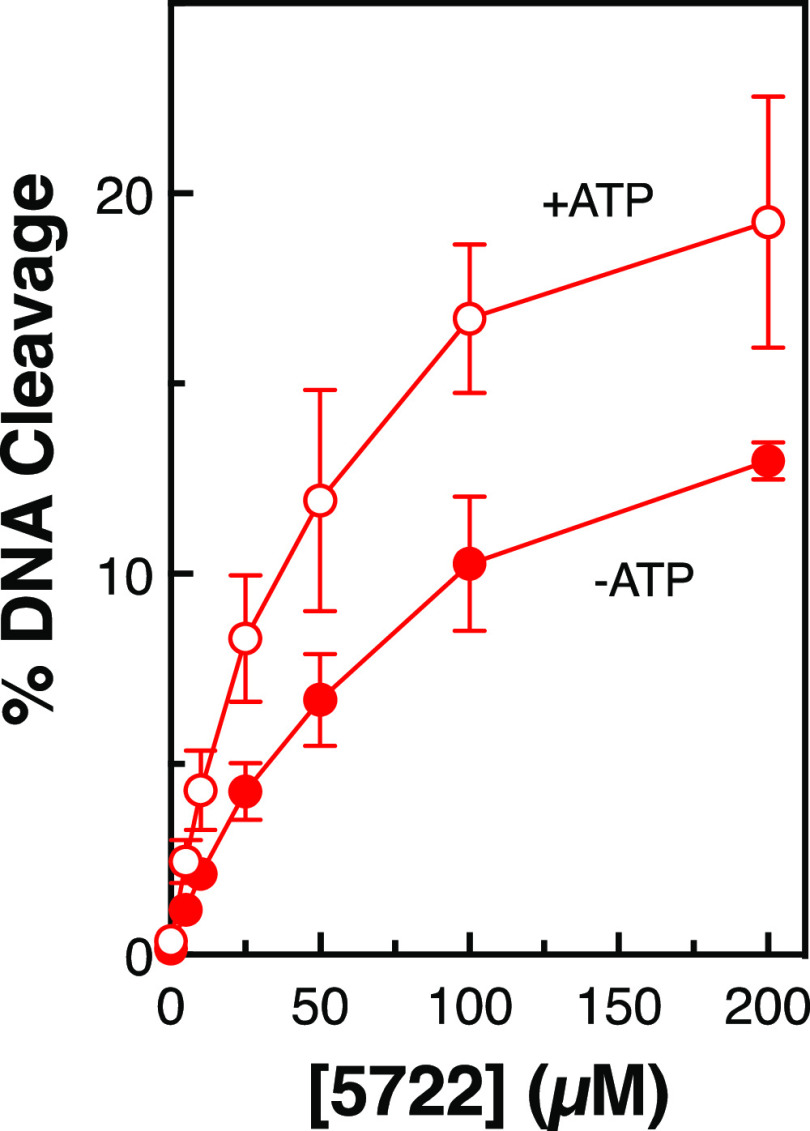
ATP enhances the stimulation of double-stranded DNA breaks mediated by *M. tuberculosis* gyrase in the presence of H3D-005722 (5722). Double-stranded DNA breaks were monitored in reactions that contained 0 (closed circles) or 1.5 mM ATP (open circles) Error bars represent the SD of at least three independent experiments.

**Figure 6:**
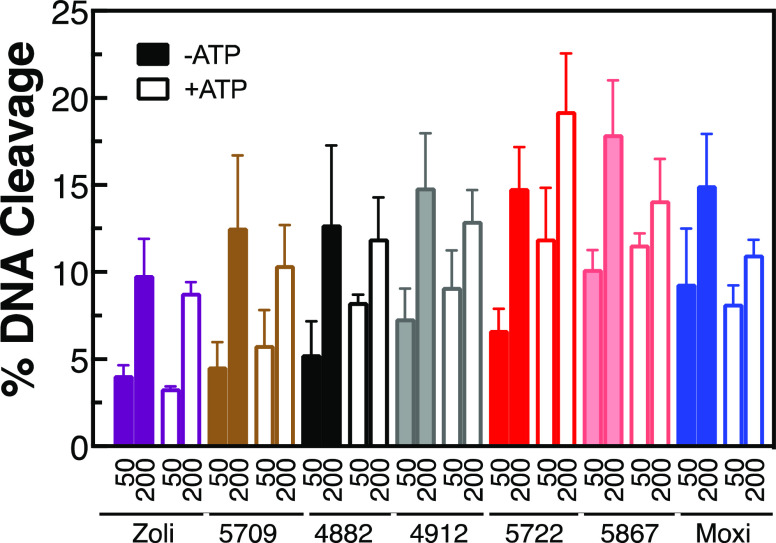
Effects of ATP on gyrase-mediated double-stranded DNA cleavage in the presence of SPTs and fluoroquinolones. Reactions containing 50 or 200 μM zoliflodacin (purple), H3D-005709 (brown), H3D-004882 (black), H3D-004912 (4912, silver), H3D-005722 (red), H3D-005867 (5867, salmon), or moxifloxacin (blue) were carried out in the absence (solid bars) or presence (open bars) of 1.5 mM ATP. Error bars represent the SD of at least three independent experiments.

The DNA cleavage reactions shown above were carried out in the absence of ATP, which is the high energy co-factor that is required for gyrase to carry out its complete catalytic DNA supercoiling reaction.^1−6^ In some cases, the inclusion of ATP has been shown to enhance the ability of drugs to induce enzyme-mediated DNA cleavage, while in others, it has diminished scission.^19,30^ The effects of ATP on the stimulation of gyrase-mediated DNA cleavage by H3D-005722 are shown in Figure 5. In the presence of ATP, the activity of the SPT was ∼1.5 to 2-fold higher at every concentration examined. Furthermore, the activities of the novel SPTs (H3D-004882, H3D-004912, H3D-005709, and H3D-005867) against the *M. tuberculosis* enzyme were also increased modestly by ATP at 50 μM SPT, although little difference was observed at 200 μM SPT (Figure 6). These findings contrast with results with zoliflodacin and moxifloxacin whose abilities to enhance DNA cleavage decreased slightly in the presence of ATP (Figure 6). Thus, in the bacterial cell, which contains (on average) millimolar concentrations of ATP,^37^ H3D-005722 is likely to be more active than zoliflodacin or moxifloxacin.

### H3D-005722 Interacts with DNA in the Cleavage–Ligation Active Site of *M. tuberculosis* Gyrase

Structural studies provide strong evidence that fluoroquinolones interact with DNA in the cleavage–ligation active site of *M. tuberculosis* gyrase.^17^ These drugs are situated between the newly generated 3′-OH and 5′-PO_4_ terminal moieties of the two scissile bonds. Because SPTs, such as fluoroquinolones, induce gyrase-mediated double-stranded DNA breaks, it is presumed that all members of this class also interact at the two scissile bonds of *M. tuberculosis* gyrase. However, there is no direct evidence to confirm this assumption. At the present time, there is a crystal structure of a cleavage complex formed with QPT-1, which is a progenitor and smaller member of the SPT drug class,^29^ and a second structure with zoliflodacin.^38^ Both structures were generated with gyrase from *Staphylococcus aureus*)*.*

**Figure 7:**
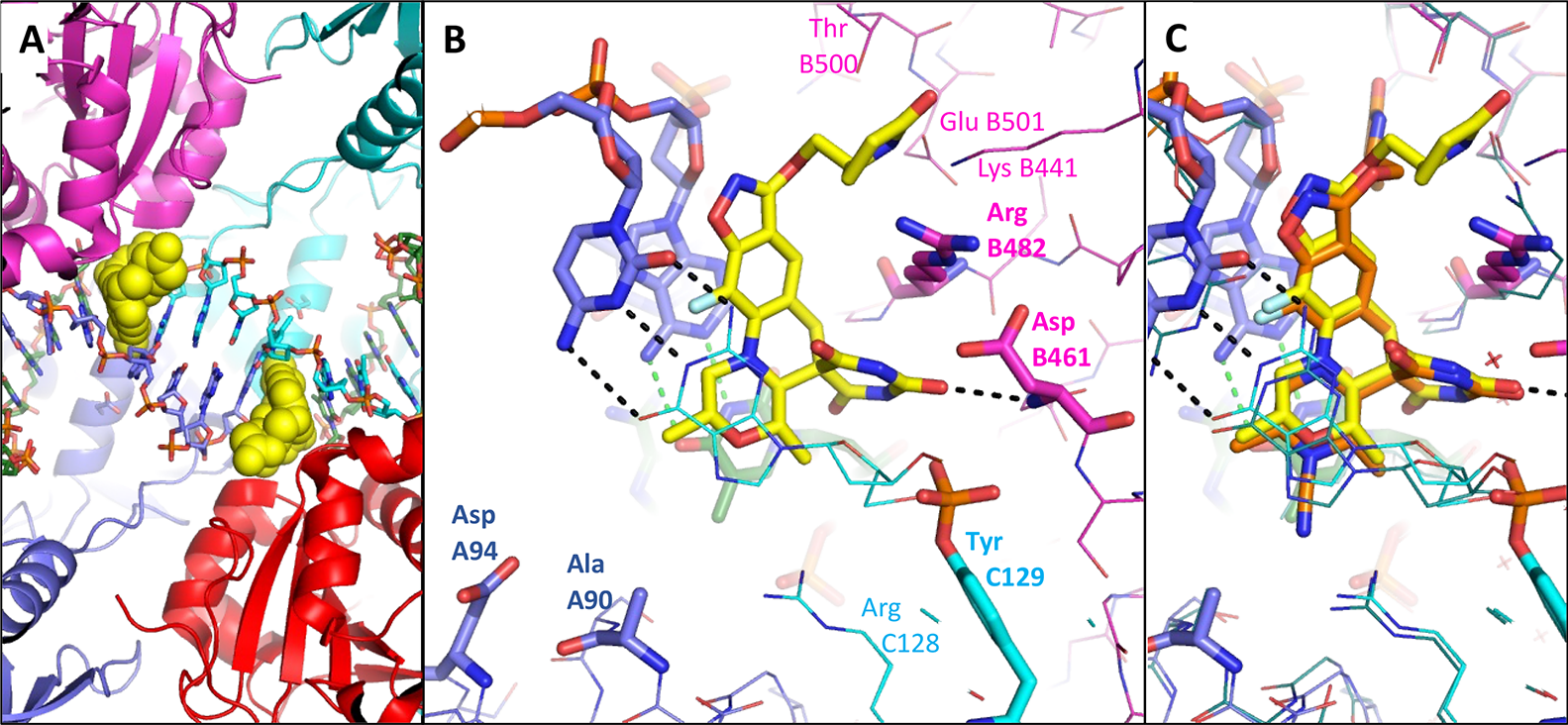
Model of SPT H3D-005722 in a DNA-cleavage complex with *M. tuberculosis* gyrase. (A) View down the twofold axis of a DNA–cleavage complex formed with *M. tuberculosis* gyrase, DNA, and two molecules of H3D-005722 (yellow atoms). The protein is shown in a cartoon with one GyrB shown in red and the other in magenta. The GyrA subunits are in cyan and dark blue. The cleaved DNA is shown in a stick representation with carbons in the DNA colored cyan when covalently attached to the cyan GyrA subunit and dark blue when linked to the dark-blue GyrA subunit, and the DNA carbons are in dark green when not covalently attached (nitrogen atoms are dark blue, oxygen atoms are red, and phosphorous atoms are orange). (B) Enlarged view of one binding site with H3D-005722 (yellow carbon atoms) shown in sticks. Some protein residues also are shown as sticks (GyrB magenta carbon atoms, dark-blue nitrogen atoms, and red oxygen atoms). Hydrogen bonds near the compound are shown as dotted lines. Note for clarity: the nucleotide covalently attached to Tyr C129 and some protein residues are shown in thinner line representations (rather than “thicker” sticks). (C) Comparison of the model of H3D-005722 with that of H3D-005867 (orange carbons) from Govender et al.^34^ Note that the only difference between the compounds is the replacement of the R_1_ oxygen in H3D-005722 with the amino-cyano group in H3D-005687. The different modeled positions of the pendant valerolactam group could be due to the flexibility in the structure, as the DNA wobbles the DNA gate of *M. tuberculosis* gyrase.

Therefore, we carried out modeling studies to help elucidate the interactions of SPTs in the active site of *M. tuberculosis* gyrase. The model shown in Figure 7 with H3D-005722 bound to the enzyme was constructed based on coordinates from the crystal structures of QPT-1 in a cleavage complex with *S. aureus* gyrase^29^ and moxifloxacin in a cleavage complex with *M. tuberculosis* gyrase,^17^ as had been carried out previously for H3D-005687.^34^ Note that the only difference between H3D-005722 and H3D-005687 is the replacement of the R_1_ morpholine oxygen of the former with the amino-cyano group of the latter. Modeling studies suggest that H3D-005722 stabilizes double-stranded DNA breaks mediated by *M. tuberculosis* gyrase by interacting at the two scissile bonds cleaved by the enzyme (Figure 7A). This presumes the binding of two SPT molecules, one at each scissile bond as seen in the crystal structures of QPT-1 and zoliflodacin bound to the *S. aureus* gyrase.^29,38^ The region surrounding the H3D-005722 R_2_ valerolactam side chain includes the GyrB residues Arg482 and Lys441 that appear to be conformationally mobile (Figure 7B). Equivalent residues in *S. aureus* gyrase (Arg 458 and Lys 417) tend to have poor electron density and vary in their positions depending on the bound inhibitor. Hence, it is likely that the conformation of the R_1_ valerolactam is ambiguous. Figure 7C shows two possible poses for the side chain using the superposition of the model of H3D-05772 and that previously published for H3D-005687 (compound **42** in the publication).^34^ The conformational mobility in the region probably accounts for the wide range of R_2_ benzisoxazole substituents (Figure 1) that are acceptable for gyrase inhibitory potency.^26,34,35^ However, the differences in the positioning of the valerolactam between the two structures could also contribute to the enhanced activity of H3D-005687 as compared to H3D-05772.

To more directly determine whether H3D-005722 functions in the DNA cleavage–ligation active site of the enzyme, competition studies were carried out using GSK000, which is an NBTI derivative with high activity against *M. tuberculosis* gyrase.^18^ In contrast to SPTs, only a single NBTI molecule binds in the cleavage complex. The left-hand side of the NBTI sits in a pocket in the DNA on the twofold axis of the complex, midway between the two DNA cleavage sites, and the right-hand side sits in a pocket on the twofold axis between the two GyrA subunits. Competition studies have demonstrated that NBTIs and fluoroquinolones that interact at the scissile bonds cannot coexist in the active site of gyrase.^18,30^

**Figure 8:**
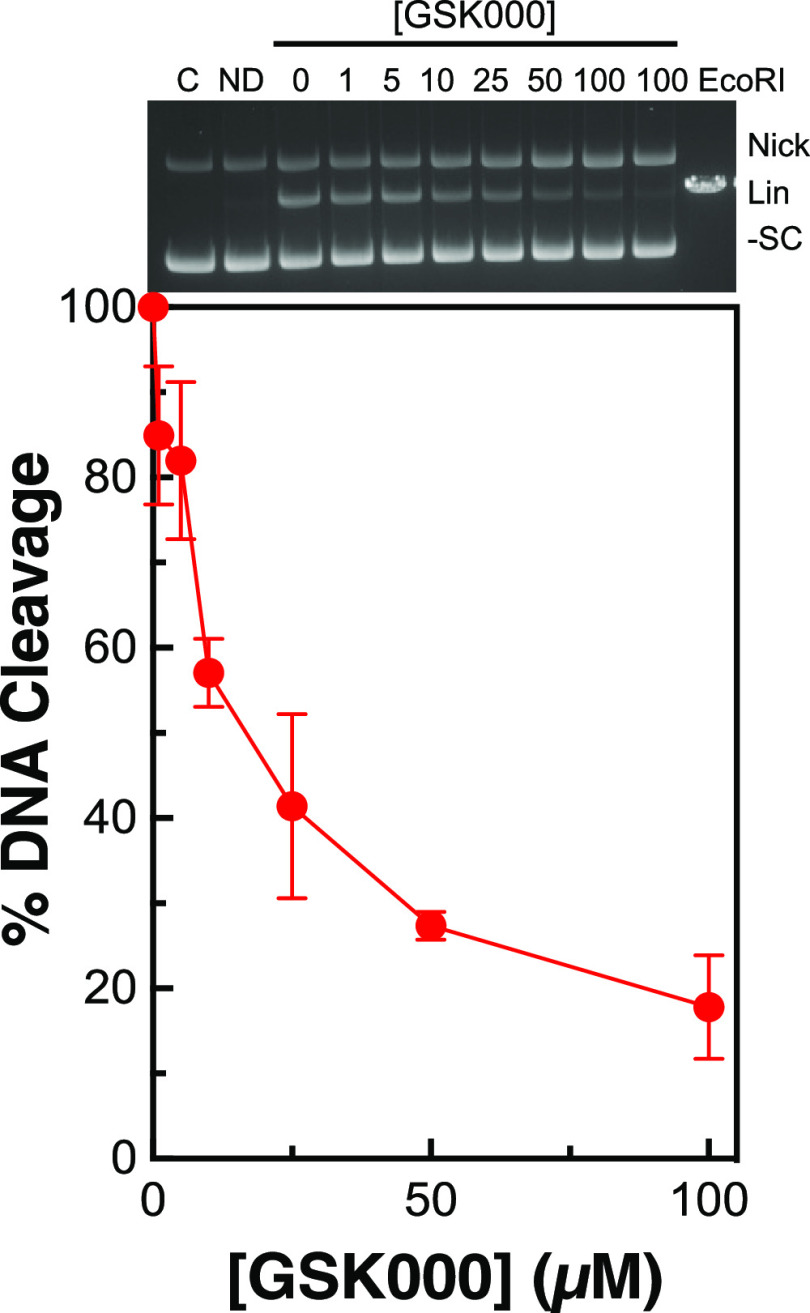
The actions of H3D-005722 and the NBTI GSK000 on *M. tuberculosis* gyrase-mediated DNA cleavage are mutually exclusive. DNA cleavage/ligation equilibria were established in the presence of 200 μM H3D-005722 plus 0–100 μM GSK000. Competition was monitored by the loss of H3D-005722-induced double-stranded DNA breaks. A representative gel is shown above the graph. The positions of negatively supercoiled (−SC), nicked (Nick), and linear (Lin) DNA are indicated. Gyrase-mediated DNA cleavage in the absence of drugs is shown (no drug - ND). Error bars represent the SD of at least three independent experiments.

The actions of NBTIs and SPTs can be distinguished because GSK000 induces only gyrase-mediated single-stranded DNA breaks,^18^ while H3D-005722 induces primarily double-stranded breaks (Figure 2). This difference was used as the basis for a competition assay to determine whether the NBTI and H3D-005722 can simultaneously act on *M. tuberculosis* gyrase. In the assay, cleavage complexes were formed in the presence of a mixture of 200 μM H3D-005722 and increasing concentrations of GSK000 (0–100 μM). Competition was monitored by the loss of double-stranded DNA breaks, which only could have been induced by the SPT. As seen in Figure 8, levels of double-stranded breaks dropped ∼80% in the presence of 100 μM GSK000, which indicates competition between the NBTI and SPT.

A caveat to this conclusion is the fact that GSK000 suppresses double-stranded DNA breaks generated by *M. tuberculosis* gyrase.^18^ Thus, it is possible that the NBTI and H3D-005722 interact with gyrase at separate sites and the apparent competition is due to this double-stranded DNA break suppression. If this were the case, GSK000 would decrease the actions of H3D-005722 at a concentration that reflects its binding to *M. tuberculosis* gyrase. In the absence of a competing compound, the concentration at which GSK000 induces one-half maximal single-stranded DNA cleavage with *M. tuberculosis* gyrase is ∼2.5 μM.^18^ However, in the presence of the SPT, considerably higher concentrations of GSK000 were required to reduce double-stranded DNA breaks by 50% (IC_50_ ≅ 17 μM; Figure 8). The reduced affinity of GSK000 for *M. tuberculosis* gyrase in the presence of H3D-005722 indicates that the decrease in double-stranded DNA breaks is due primarily to a competition between the SPT and the NBTI. Taken together, these findings provide evidence that GSK000 and H3D-005722 cannot co-exist in the cleavage complex, which suggests that the SPT exerts its actions on *M. tuberculosis* gyrase by interacting in the active site of the enzyme.

### SPTs Maintain Activity against *M. tuberculosis* Gyrase Enzymes Carrying Common Mutations Associated with Fluoroquinolone Resistance

In a previous study, an SPT similar to H3D-005722 maintained activity against *M. tuberculosis* cells that carried the gyrase mutation GyrA^A90V^, which elicits fluoroquinolone resistance.^35^ However, the effects of SPTs on the DNA cleavage activity of *M. tuberculosis* gyrase enzymes that harbor this and other common fluoroquinolone resistance mutations have not been examined. Therefore, the effects of SPTs on DNA scission mediated by gyrase enzymes that harbor the GyrA^A90V^, GyrA^D94G^, or GyrA^D94H^ mutation, three of the most common mutations associated with fluoroquinolone-resistance in *M. tuberculosis*,^20^ were determined.

**Figure 9:**
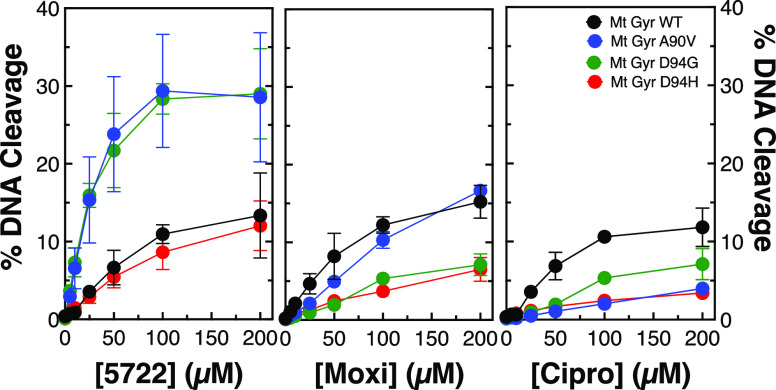
H3D-005722 overcomes the presence of common fluoroquinolone resistance mutations in *M. tuberculosis* gyrase. Double-stranded DNA cleavage reactions mediated by WT (black) gyrase or enzymes harboring the GyrA^A90V^ (A90V, blue), GyrA^D94G^ (D94G, green), or GyrA^D94H^ (D94H, red) mutations in the presence of H3D-005722 (5722, left panel), moxifloxacin (Moxi, center panel), or ciprofloxacin (Cipro, right panel) are shown. Error bars represent the SD of at least three independent experiments.

**Figure 10:**
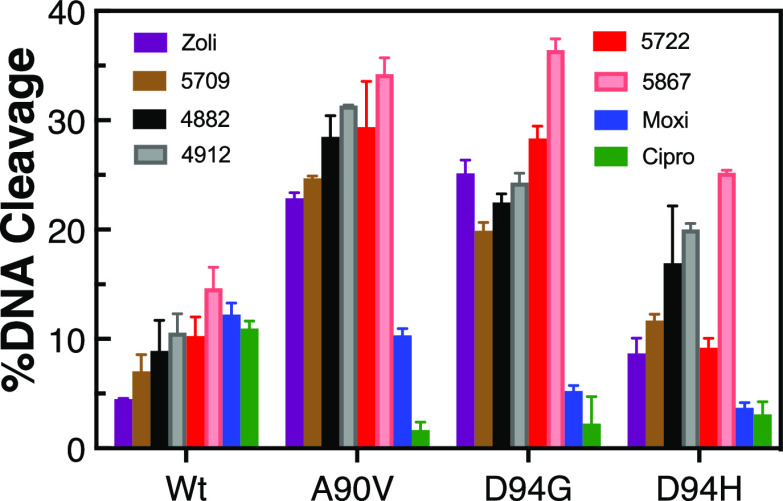
Novel SPTs overcome the presence of common fluoroquinolone resistance mutations in *M. tuberculosis* gyrase. Double-stranded DNA cleavage reactions mediated by WT gyrase or enzymes harboring the GyrA^A90V^ (A90V), GyrA^D94G^ (D94G), or GyrA^D94H^ (D94H) mutations in the presence of 200 μM zoliflodacin (Zoli, purple), H3D-005709 (5709, brown), H3D-004882 (4882, black), H3D-004912, (4912, silver), H3D-05722, (5722, red), H3D-005867 (5867, salmon), moxifloxacin (Moxi, blue), and ciprofloxacin (Cipro, green) are shown. Error bars represent the SD of at least three independent experiments. In the absence of compounds, less than 0.3% of the DNA was cleaved by all the enzymes.

As seen in Figure 9 (middle and right panels), the three mutant enzymes displayed resistance toward moxifloxacin and ciprofloxacin. In contrast, H3D-005722 maintained the ability to induce double-stranded DNA cleavage by the mutant gyrase enzymes (Figure 9, left panel). Although the activity of the SPT against GyrA^D94H^ gyrase approximated that of the wild-type enzyme, H3D-005722 induced considerably higher levels (approximately three-fold) of DNA cleavage with the GyrA^A90V^ and GyrA^D94G^ mutant enzymes. Similar results were observed for all the SPTs utilized in the present study (Figure 10). In all cases, the compounds displayed much higher levels of DNA cleavage with the GyrA^A90V^ and GyrA^D94G^ enzymes and at least wild-type activity against GyrA^D94H^ gyrase. Modeling studies indicate that GyrA residues A90 and D94 are not proximal to the bound SPT (Figure 7B). Thus, at the present time, there is not an obvious explanation for the enhanced activity of SPTs against fluoroquinolone-resistant mutations at these amino acid residues. However, the results of DNA cleavage assays demonstrate that H3D-005722 and related SPTs overcome the most common causes of target-mediated fluoroquinolone resistance in tuberculosis, at least at the enzyme level.

### Effects of SPTs on DNA Cleavage Mediated by Human Topoisomerase IIα

**Figure 11:**
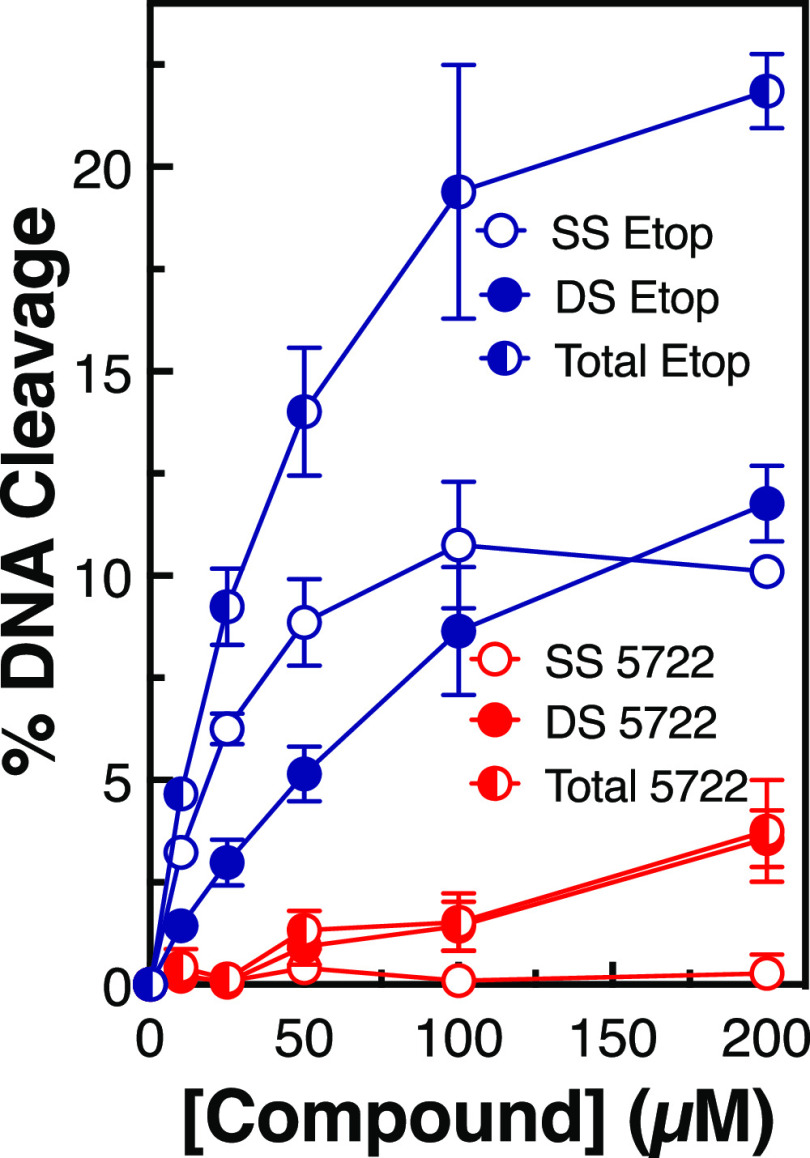
H3D-005722 (red) enhances double-stranded (DS, closed circles) DNA cleavage mediated by human topoisomerase IIα at a low level compared to the well-known human topoisomerase IIα poison, etoposide (dark blue). H3D-005722 does not enhance single-stranded (SS, open circles) DNA cleavage, while etoposide generates higher levels of SS DNA cleavage than DS DNA cleavage. Total (DS + SS, half-solid circles) DNA cleavage is also shown. Error bars represent the SD of at least three independent experiments.

**Figure 12:**
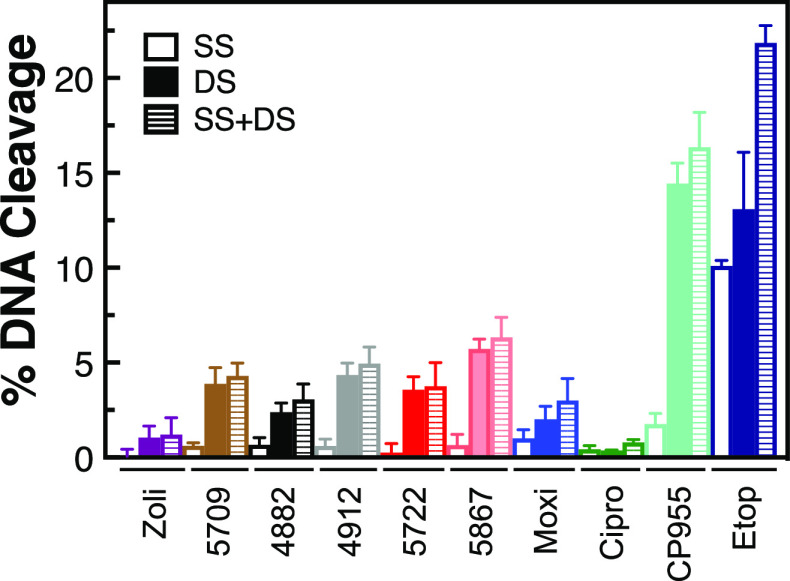
SPTs enhance double-stranded (DS, solid bars) DNA cleavage mediated by human topoisomerase IIα at low levels and do not enhance single-stranded (SS, open bars) DNA cleavage at 200 μM zoliflodacin (Zoli, purple), H3D-005709 (5709, brown), H3D-004882 (4882, black), H3D-004912, (4912, silver), H3D-05722, (5722, red), or H3D-005867 (5867, salmon). The fluoroquinolones, moxifloxacin (Moxi, blue) and ciprofloxacin (Cipro, green), also induce low levels of DNA cleavage, while the human topoisomerase II poisons, CP-115,955 (CP955, light green) and etoposide (dark blue), generate high levels of DS and SS DNA cleavage (total DS + SS DNA cleavage, hatched bars). Error bars represent the SD of at least three independent experiments.

Because gyrase and topoisomerase IIα are homologous enzymes, some antibacterial drug classes have the capacity to cross over into mammalian systems.^39−41^ Therefore, the effects of H3D-005722 and related SPTs on the DNA cleavage activity of human topoisomerase IIα were assessed (Figures 11 and 12). At 200 μM, H3D-005722 induced a modest rise in double-stranded DNA breaks generated by topoisomerase IIα. However, virtually no increase in either double- or single-stranded cleavage was observed at any lower concentrations. Even at the highest concentration of H3D-005722 examined (200 μM), the rise in total DNA cleavage (double- and single-stranded) was dwarfed by that of etoposide, a topoisomerase II-targeted anticancer drug. At 200 μM, all the SPT and fluoroquinolone antibacterials examined displayed modest activity against human topoisomerase IIα compared to etoposide and CP-115,955,^41^ which is a fluoroquinolone designed to have high activity against eukaryotic type II topoisomerases (Figure 12). Therefore, these SPTs maintain their potential for development as antibacterial drugs.

## Discussion

There is a need for the development of novel antitubercular agents to address rising drug resistance in this disease. Like the fluoroquinolones, SPTs act through gyrase, which is a validated drug target in *M. tuberculosis*.^10,11^ Results of the present study indicate that H3D-005722 and related SPTs display high activity against *M. tuberculosis* gyrase and increase levels of enzyme-mediated double-stranded DNA cleavage. The activities of the SPTs examined were similar to those of moxifloxacin and ciprofloxacin and greater than that of zoliflodacin, a clinically advanced SPT. Finally, H3D-005722 and other novel SPTs overcome the most common mutations in gyrase that cause fluoroquinolone resistance and, in most cases, were more active against the mutant enzymes than the wild-type gyrase.

All of the compounds that were assessed in the present study inhibit the growth of cultured H37Rv *M. tuberculosis* cells with minimal inhibitor concentrations (MICs) in the micromolar range.^34^ The order of efficacy was H3D-005867 > H3D-005722 > H3D-005709 > H3D-004882 > zoliflodacin > H3D-004912. This order is similar but not identical to the maximal levels of *M. tuberculosis* gyrase double-stranded DNA cleavage induced by these compounds, H3D-005867 > H3D-004912 > H3D-005722 > H3D-005709 ≈ H3D-004882 > zoliflodacin (Figure 2). The most notable difference is that H3D-004912, which was the second most efficacious SPT with regard to DNA cleavage was the least inhibitory with regard to cell growth. The reasons that underlie this discrepancy are not known but may reflect cellular uptake, efflux, or metabolism. It is also notable that the range of MIC values is considerably lower (0.5–7.8 μM)^34^ than the concentrations used in the present study. However, the relationship between concentrations of compounds used in cellular assays and the actual levels that accumulate in cells is unclear. Furthermore, the number of DNA strand breaks required to kill *M. tuberculosis* cells is not known.

Finally, SPTs (like fluoroquinolones and NBTIs) have two effects on *M. tuberculosis* gyrase. They increase levels of enzyme-mediated DNA cleavage and they inhibit overall catalytic activity.^16,18,34^ It is generally assumed that drugs that enhance gyrase-mediated DNA cleavage (i.e., poisons) kill cells primarily by inducing breaks in the bacterial genome.^2,4−6,9^ In this case, the higher the cellular concentration of gyrase, the greater the number of DNA breaks, and the more lethal the drug. However, a recent report indicated that *M. tuberculosis* cells that were gyrase hypomorphs displayed hypersensitivity to SPTs.^34^ This finding suggests that SPTs are also capable of killing cells by inhibiting the essential catalytic activities (removing positive DNA supercoils that accumulate ahead of replication forks and introducing negative supercoils into the genome) of gyrase.^4,7,8^ Our findings, along with the previous cellular and in vitro studies,^34^ suggest a model in which SPTs (and other gyrase-targeted drugs) may be able to kill *M. tuberculosis* cells in a bimodal fashion. At high gyrase concentrations, drugs act as “poisons” that fragment the genome. Conversely, at low gyrase concentrations, drugs act as catalytic inhibitors that rob cells of a critical enzyme activity. This “bimodal model” of cell kill may allow SPTs to act under a variety of growth conditions. Taken together, these findings further support the potential of novel SPT derivatives as antitubercular drugs.

## Materials and Methods

### Enzymes and Materials

Wild-type *M. tuberculosis* gyrase subunits (GyrA and GyrB) and fluoroquinolone-resistant GyrA mutants (GyrA^A90V^, GyrA^D94H^, and GyrA^D94G^) were expressed and purified as described previously^16^ and were stored at −80 °C. Human topoisomerase IIα was expressed and purified from *Saccharomyces cerevisiae*(42,43) and was stored in liquid N_2_.

Negatively supercoiled pBR322 DNA was prepared from *E. coli* using a Plasmid Mega Kit (Qiagen) as described by the manufacturer.

The SPTs H3D-004882, H3D-004912, H3D-005709, H3D-005722, and H3D-005867 were synthesized as described previously by Govender et al.^34^ In that paper, H3D-004882 and H3D-005722 were referred to as compounds **8** and **23,** respectively. Zoliflodacin was obtained from MedChemExpress, moxifloxacin from LKT Laboratories, and ciprofloxacin and etoposide from Sigma-Aldrich (Millipore Sigma). The SPTs, moxifloxacin, etoposide, and the NBTI derivative GSK000 (gift from Monica Cacho) were stored at −20 °C as 20 mM stock solutions in 100% dimethyl sulfoxide (DMSO). H3D-005722 working concentrations (5–200 μM) were not soluble in 10% DMSO but were soluble in 40% DMSO. Consequently, all drug dilutions for the SPTs and fluoroquinolones were in 40% DMSO. The final concentration of DMSO in reaction mixtures was 4%. Ciprofloxacin and the fluoroquinolone CP-115,955 (gift from Robert Kerns) were stored as 40 mM stock solutions in 0.1 N NaOH and stored at −20 °C. Prior to their use in assays, these fluoroquinolones were diluted fivefold into 10 mM Tris–HCl (pH 7.9).

### DNA Cleavage

DNA cleavage reactions were based on the procedure of Aldred et al.^16^ Reactions were carried out in the presence or absence of SPTs or fluoroquinolones and contained 100 nM wild-type or fluoroquinolone-resistant mutant (GyrA^A90V^, GyrA^D94H^, and GyrA^D94G^) gyrase (1.5:1 GyrA:GyrB ratio) and 10 nM negatively supercoiled pBR322 in a total volume of 20 μL of gyrase cleavage buffer [10 mM Tris–HCl (pH 7.5), 40 mM KCl, 6 mM MgCl_2_, 0.1 mg/mL bovine serum albumin, and 10% glycerol]. In some cases, 1.5 mM ATP was included in reaction mixtures. Unless stated otherwise, reactions were incubated at 37 °C for 10 min. Enzyme–DNA cleavage complexes were trapped by adding 2 μL of 4% sodium dodecyl sulfate (SDS) followed by 1 μL of 375 mM Na_2_EDTA and 2 μL of 0.8 mg/mL Proteinase K (Sigma Aldrich). Reaction mixtures were incubated at 45 °C for 30 min to digest the enzyme. Samples were mixed with 2 μL of 60% sucrose, 10 mM Tris–HCl (pH 7.9), 0.5% bromophenol blue, and 0.5% xylene cyanol FF and incubated at 45 °C for 2 min before loading onto 1% agarose gels. Reaction products were subjected to electrophoresis in 40 mM Tris–acetate (pH 8.3) and 2 mM EDTA containing 0.5 μg/mL ethidium bromide. DNA bands were visualized with medium-range ultraviolet light and quantified using an Alpha Innotech digital imaging system. DNA single or double-stranded cleavage was monitored by the conversion of negatively supercoiled plasmid to nicked or linear molecules, respectively, and quantified in comparison to a control reaction in which an equal amount of DNA was digested by EcoRI (New England BioLabs).

In reactions that examined the effects of compounds on the DNA cleavage activity of human topoisomerase IIα, the procedure of Fortune and Osheroff was employed.^44^ Reaction mixtures contained 0–200 μM H3D-005722 or etoposide, 110 nM topoisomerase IIα, and 10 nM negatively supercoiled pBR322 in a total volume of 20 μL of 10 mM Tris–HCl (pH 7.9), 100 mM KCl, 0.1 mM EDTA, 5 mM MgCl_2,_ and 2.5% (*v*/*v*) glycerol. Assay mixtures were incubated at 37 °C for 6 min. Reactions were terminated, and products were analyzed as described above. Additional experiments with topoisomerase IIα compared DNA cleavage induced by 200 μM zoliflodacin, the rest of the novel SPT series, moxifloxacin, ciprofloxacin, and CP-115,955,^41^ which is a fluoroquinolone developed for its high activity against eukaryotic type II topoisomerases.

### DNA Ligation

DNA ligation assays were carried out in the absence or presence of H3D-005722, moxifloxacin, or ciprofloxacin following the procedure of Gibson et al.^18^ Reaction mixtures (20 μL) contained 100 nM wild-type *M. tuberculosis* gyrase and 10 nM negatively supercoiled pBR322 in gyrase cleavage buffer. In experiments carried out in the absence of a drug, MgCl_2_ in the cleavage buffer was replaced with 6 mM CaCl_2_ to increase baseline levels of DNA cleavage. DNA cleavage–religation equilibria were established at 37 °C for 10 min. Ligation was initiated by shifting the temperature from 37 to 75 °C. Reactions were stopped by the addition of 2 μL of 4% SDS followed by 1 μL of 375 mM EDTA (pH 8.0). Samples were digested with Proteinase K, processed, and visualized as described above. Levels of double-stranded DNA cleavage were set to 100 at time = 0 s, and ligation was assessed by the loss of the linear reaction product over time.

### Molecular Modeling

To guide the placement of H3D-005722 in the cleavage complex, structures were modeled using Coot^45−48^ and Maestro (Schrödinger Release 2019-1: Maestro, Schrödinger, LLC, New York, NY, 2019). The placement of H3D-005722 in a ternary complex with *M. tuberculosis* gyrase was based on a crystal structure of QPT-1 in a cleavage complex with *Staphylococcus aureus* gyrase^29^ [Protein Data Bank (PDB) code: 5CDM] and a crystal structure of moxifloxacin in a cleavage complex with *M. tuberculosis* gyrase^17^ (PDB code 5BS8). Other related structures were examined and informed the modeling.
